# Development of Different Kinds of Electrocatalyst for the Electrochemical Reduction of Carbon Dioxide Reactions: An Overview

**DOI:** 10.3390/molecules28207016

**Published:** 2023-10-10

**Authors:** Tse-Wei Chen, Shen-Ming Chen, Ganesan Anushya, Ramanujam Kannan, Abdullah G. Al-Sehemi, Saranvignesh Alargarsamy, Pandi Gajendran, Rasu Ramachandran

**Affiliations:** 1 Department of Materials, Imperial College London, London SW7 2AZ, UK; t.chen19@imperial.ac.uk; 2 Department of Chemical Engineering and Biotechnology, National Taipei University of Technology, Taipei 10608, Taiwan; saranvignesh1507@gmail.com; 3 Department of Physics, St. Joseph College of Engineering, Sriperumbudur, Chennai 602 117, India; g.anushya7@gmail.com; 4 Department of Chemistry, Sri Kumara Gurupara Swamigal Arts College (Affiliated to Manomaniam Sundaranar University), Srivaikuntam, Thoothukudi 628 619, India; tgrk2013@gmail.com; 5 Research Center for Advanced Materials Science (RCAMS), King Khalid University, Abha 61413, Saudi Arabia; agsehemi@kku.edu.sa; 6 Department of Chemistry, College of Science, King Khalid University, Abha 61413, Saudi Arabia; 7 Department of Chemistry, The Madura College (Affiliated to Madurai Kamaraj University), Vidya Nagar, Madurai 625 011, India; haigaja78@yahoo.com

**Keywords:** electrocatalysts, carbon dioxide reduction, carbon monoxide, formate, methanol

## Abstract

Significant advancements have been made in the development of CO_2_ reduction processes for applications such as electrosynthesis, energy storage, and environmental remediation. Several materials have demonstrated great potential in achieving high activity and selectivity for the desired reduction products. Nevertheless, these advancements have primarily been limited to small-scale laboratory settings, and the considerable technical obstacles associated with large-scale CO_2_ reduction have not received sufficient attention. Many of the researchers have been faced with persistent challenges in the catalytic process, primarily stemming from the low Faraday efficiency, high overpotential, and low limiting current density observed in the production of the desired target product. The highlighted materials possess the capability to transform CO_2_ into various oxygenates, including ethanol, methanol, and formates, as well as hydrocarbons such as methane and ethane. A comprehensive summary of the recent research progress on these discussed types of electrocatalysts is provided, highlighting the detailed examination of their electrocatalytic activity enhancement strategies. This serves as a valuable reference for the development of highly efficient electrocatalysts with different orientations. This review encompasses the latest developments in catalyst materials and cell designs, presenting the leading materials utilized for the conversion of CO_2_ into various valuable products. Corresponding designs of cells and reactors are also included to provide a comprehensive overview of the advancements in this field.

## 1. Introduction

Nanoparticle catalysts frequently exhibit improved performance in comparison to larger surfaces due to their increased surface-to-volume ratio and the exposure of new active sites, leading to heightened intrinsic activity. Moreover, having precise control over the shape and size of nanoparticles is advantageous for comprehending trends in catalytic activity and designing effective catalyst designs. Despite this, there have been concerns about the preservation of nanoparticles original morphologies during catalytic reactions [1]. Since the advent of the industrial revolution, there has been a continuous upward trajectory in technological progress globally. Simultaneously, there has been an escalating reliance on the utilization of fossil fuels such as coal, petroleum, and natural gas as the primary energy source to sustain our societies and economies. However, the depletion of these non-renewable energy sources is progressively intensifying, leading to a multitude of challenges [2,3].

Conversely, the combination of the aforementioned progress and the rapid expansion of the population has led to a surge in energy requirements. However, given the finite reserves of fossil fuels, which are non-renewable resources, we are gradually approaching a worsening energy crisis. The limited availability of these resources poses a significant challenge in meeting the escalating demand for energy. The electrochemical conversion of carbon dioxide (CO_2_) into valuable chemicals and fuels has garnered significant attention due to its favorable characteristics, including the use of environmentally friendly electrolytes, the ability to fine-tune product formation, the potential integration of renewable energy sources, and mild reaction conditions. However, the electrochemical CO_2_ reduction reaction (CO_2_RR) faces numerous challenges, such as high energy barriers, the occurrence of multiple concurrent reactions, and competition from the hydrogen evolution reaction (HER). Consequently, the CO_2_RR yields a diverse range of chemical products, including carbon monoxide (CO), formate (HCOO), methane (CH_4_), methanol (CH_3_OH), ethane (C_2_H_6_), ethylene (C_2_H_4_), and ethanol (C_2_H_5_OH), with the inevitable formation of hydrogen (H_2_) in aqueous electrolytes [4,5,6,7,8,9,10].

Liu et al. [11] present a simple anodic corrosion method to synthesize oxygen-richCuO nanoplate arrays that undergo evolution during electrocatalysis, forming Cu/Cu_2_O interfaces. The catalyst demonstrates impressive performance with 84.5% Faradaic efficiency for C_2_H_4_ production, stable electrolysis for ~55 h, and 27.6% ethylene energy efficiency at 200 mA cm^−2^. Mechanistic analyses highlight the importance of stable nanostructures and enhanced *OCCOH intermediate adsorption for selective and prolonged C_2_H_4_ generation. Co-electrolysis of CO_2_ with small amounts of O_2_ generates surface hydroxyl species, which dramatically enhance the activity of copper catalyzed CO_2_ electroreduction. Although multiple oxidative treatments have improved copper catalyst performance, the underlying mechanism for this enhancement remains controversial. Copper is the only monometallic electrocatalyst capable of converting CO_2_ into valuable hydrocarbons and oxygenates, yet it exhibits subpar selectivity and activity [12].

Efforts in the field of research are currently focused on acquiring a catalyst that meets the criteria for large-scale electrochemical conversion of CO_2_. The objective is to achieve enhanced efficiency, minimal overpotential, and heightened selectivity towards specific products. This pursuit aims to establish CO_2_ electrochemical reduction as a sustainable method for storing chemical energy in the form of liquid fuels and valuable chemical feed stocks [13,14,15,16]. A wide variety of electrode materials have been used to investigate the CO_2_ reduction reaction (CO_2_RR). These materials include carbon-based materials (such as carbon [17], graphene oxide [18], and conducting polymers [19]), metal oxides [20], metal–organic frameworks (MOFs) [21], metals [22], transition metals [23], MXenes [24] and free-standing materials [25]. Each material has unique properties that make it suitable for studying the CO_2_RR. Carbon-based materials have a large surface area and good electrical conductivity, facilitating efficient electron transfer. Metal oxides exhibit diverse catalytic properties due to metal cations and oxygen vacancies. MOFs offer tunable porosity and different active sites, allowing precise control over the CO_2_RR. Metals and transition metals have distinct catalytic properties because of their ability to undergo redox reactions. MXenes demonstrate high conductivity and surface reactivity, making them promising for CO_2_RR studies. Free-standing materials, without substrate support, enhance accessibility to active sites and reduce mass transport limitations. The remarkable progress in the development of various electrode materials for the electrochemical reduction of carbon dioxide has led to the production of valuable products such as ethylene, format, carbon monoxide, and methanol. This summary of progress is presented in Table 1.

The objective of this review is to provide a comprehensive overview of recent advancements in the utilization of nanostructure-based electrocatalysts for promoting the electrochemical reduction of carbon dioxide (CO_2_RR). Initially, it delves into the basic principles of the CO_2_RR process and subsequently focuses on a selection of representative nanocatalysts based on different kinds of electrode materials in order to elucidate the relationship between their structure and properties, as well as their catalytic mechanisms in CO_2_RR. In the future, different kinds of electrode materials hold great promise as a potential candidate for commercial carbon dioxide reduction reaction (CO_2_RR) catalysts due to its straightforward synthesis method, gentle electrolysis conditions, and extended stability during electrolysis. The combination of these factors makes it an attractive option for large-scale applications in CO_2_RR.

## 2. Carbon-Based Electrode Materials

For years, various carbon-based materials such as glassy carbon, graphite, diamond doped with boron (BDD), carbon black, carbon nanofiber, carbon tubes (CNTs), and more recently, graphene, have found widespread use as electrodes in both fundamental breakthroughs and practical applications within the fields of electrochemistry and electroanalysis. In comparison to conventional solid metal electrodes, these carbon materials possess unique characteristics that set them apart. They exhibit long-term stability, a wide range of surface chemistry, and stable carbon bonds with various surface modifiers [31,32,33]. The captivating appeal of porous carbon spheres has sparked immense fascination due to their regulated porous formations, expansive surface area, functional surface properties, and exceptional electrical conductivity [34]. On the other hand, the extensive investigations have been conducted on biomass-derived nanoporous carbon materials as electrocatalysts for the oxygen reduction reaction (ORR), owing to their abundant and renewable nature. This type of materials possesses ample hierarchical architectures and substantial surface areas, both of which serve vital functions in various electrochemical applications [35,36]. The increasing focus on metal-free carbon materials is attributed to their exceptional characteristics, including cost-effectiveness, substantial surface area, remarkable conductivity, outstanding stability, and elevated electrochemical reactivity. These distinctive properties have garnered significant interest.

Reprinted with permission from [26]. The American Chemical Society Washington, DC, USA, 2019.

Pengfei Yao et al. developed a nitrogen-doped nanoporous carbon sheet with substantial surface area, pore volume, and pyridinic nitrogen content. This material demonstrated significantly enhanced selectivity for CO, reaching an impressive 90% at a remarkably low overpotential of −0.31 V, surpassing the performance of most nitrogen-doped carbon materials. The calcination temperature exerted a profound influence on the porous structure and the types of nitrogen species present in the catalyst.

**Figure 1:**
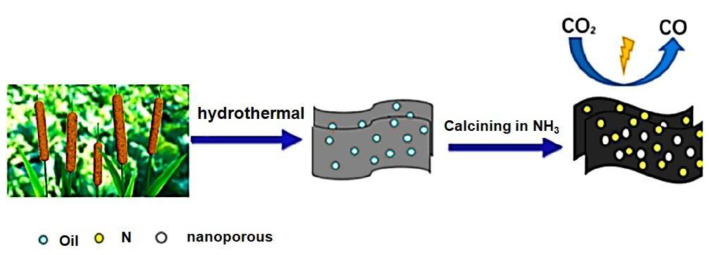
A schematic depiction of the progress made in developing nitrogen-doped nanoporous carbon materials for the electrochemical reduction of carbon dioxide.

**Figure 2:**
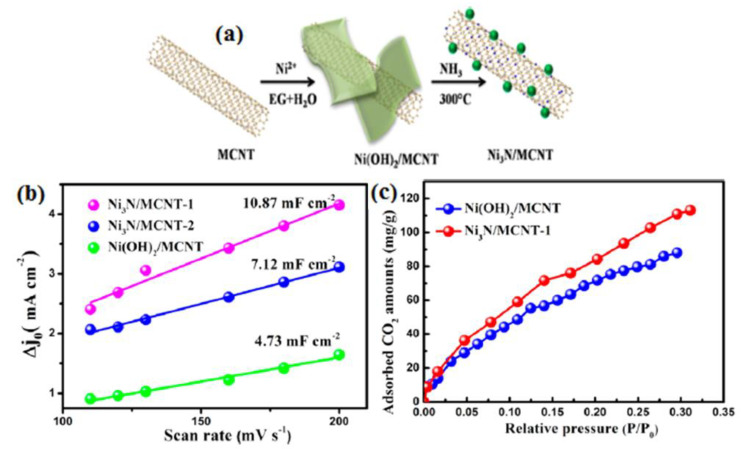
(**a**) Development of Ni_3_N/MCNT, (**b**) charging current densities vs. cyclic voltammetry sweep rates, and (**c**) CO_2_ adsorption isotherms for Ni(OH)_2_/MCNT and Ni_3_N/MCNT. Reprinted with permission from [27]. The American Chemical Society Washington, DC, USA, 2019.

Notably, the involvement of pyridinic nitrogen species played a crucial role in dictating the catalytic performance (Figure 1) [26]. Carbon materials are often employed as electrocatalyst supports due to their conductivity and substantial surface area. In this study, the electrocatalytic performance of glassy carbon (GC) supported pure graphite (PG), graphene oxide (GO), and carbon nanotubes (CNT) was evaluated for the electrochemical reduction of CO_2_ (CO_2_RR) in an aqueous solution. Notably, all three carbon materials, namely PG, GO, and CNT dispersed on GC, displayed considerable electrochemical activity, which can be attributed to the presence of impurities such as Ni, Fe, Mn, and Cu. The electrochemical activity and methane production through CO_2_RR were significantly enhanced by electrochemically depositing copper on graphene oxide (GO) and pure graphite (PG) supported on glassy carbon (GC), as well as on GC alone. Notably, GO exhibited the ability to remove nearly all catalytically relevant metals [37].The electrocatalytic reduction of CO_2_ under acidic conditions can be facilitated by Ni3N/MCNT nanocomposites, which exhibit impressive current density and CO selectivity at moderate potentials. This research provides a promising opportunity to develop efficient catalysts for the electrochemical reduction of CO_2_, allowing for the possibility of designing catalysts with superior performance (Figure 2) [27]. A cathode catalyst for CO_2_ electro reduction has been developed by Xiaofei Hu et al. [38], using nitrogen (N)-doped nanocarbon derived from metal–organic frameworks. This N-doped nanocarbon, characterized by its porous and highly conductive nature, exhibits excellent capability for CO_2_ uptake and binding. As a result, the electroreduction of CO_2_ is significantly accelerated, leading to the formation of discharged products with a thin, sheet-like structure (200 nm thickness) that can be easily decomposed during charging. This design enables reduced discharge/charge overpotential, high discharge capacity (>10,000 mAh g^−1^), long cycle life, and a high energy density of 180.

**Figure 3:**
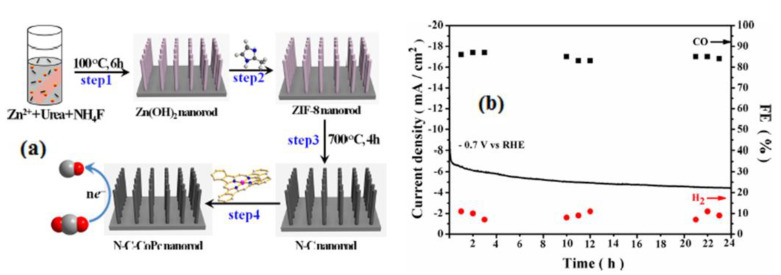
(**a**) Scheme of the process of N-C-CoPc NR and (**b**) cyclic stability test of N-C-CoPc NR at −0.7 V vs. RHE and the corresponding FEs of CO and H_2_. Reprinted with permission from [28]. The American Chemical Society Washington, DC, USA, 2020.

Whkg^−1^ in pouch cells at 50 °C. Using a hydrothermal method, Hong-Lin Zhu has employed a synergistic approach to create an electrode composed of N-doped porous carbon nanoarrays with anchored cobalt phthalocyanine (N-C-CoPc NR) electrocatalyst (Figure 3a). The N-C-CoPc NR catalyst demonstrates remarkable performance, exhibiting a high overall current density of 30 mA/cm^2^ and a significantly lower overpotential of 180 mV for the electrochemical reduction of CO_2_ to CO in a 0.1 M KHCO_3_ electrolyte. Moreover, the maximum Faradaic efficiency for CO achieved at −0.7 V vs. RHE is 85.3%, accompanied by excellent stability over time (Figure 3b) [28]. Advantages of using carbon-based electrode materials for CO_2_ reduction include their abundance and cost-effectiveness, excellent electrical conductivity, tunable surface chemistry for enhanced catalytic activity, and stability for long-term use. However, carbon materials often exhibit lower catalytic activity compared to metals, resulting in slower reactions and reduced selectivity. They can generate multiple products, making high selectivity challenging. Carbon surfaces are susceptible to contamination, which can affect performance over time, and they may have limited active sites, diminishing overall catalytic activity.

## 3. Graphene-Based Electrode Materials

Graphene, a lattice of sp^2^-bonded carbon arranged in a honeycomb pattern, has garnered significant interest as an electrode in various applications, including electrochemical devices, fuel cells, and lithium-ion batteries. Its remarkable properties, such as a vast surface area, exceptional carrier mobility, superior thermal conductivity, and excellent current and heat conduction, have contributed to its growing prominence. This has led to extensive research and exploration of graphene’s potential in these fields. Graphene has been employed as a substrate for active phases like nanoparticles or nanosheets, serving as a support material. Through its electronic and structural coupling effects, graphene has the potential to amplify charge transport and expedite CO_2_ conversion. This characteristic makes graphene a promising candidate for enhancing the efficiency of CO_2_ conversion processes [39,40,41,42,43]. While pristine graphene or carbon nanotubes (CNTs) do not display any activity in electroreducing CO_2_, remarkable catalytic efficiency has been witnessed in N-doped carbon materials such as N-doped graphene, N-doped CNTs, N-doped carbon nanofibers, and N-doped activated carbon. N-doped carbon materials exhibit superior selectivity towards CO at lower overpotentials compared to numerous metal catalysts, as reported by previous studies [44]. In a study conducted by Kumar et al., it was revealed that N-doped carbon nanofiber electrodes demonstrated the ability to convert CO_2_ into CO with a Faradaic efficiency exceeding 78% at an applied potential of −0.6 V vs. RHE [45].

**Figure 4:**
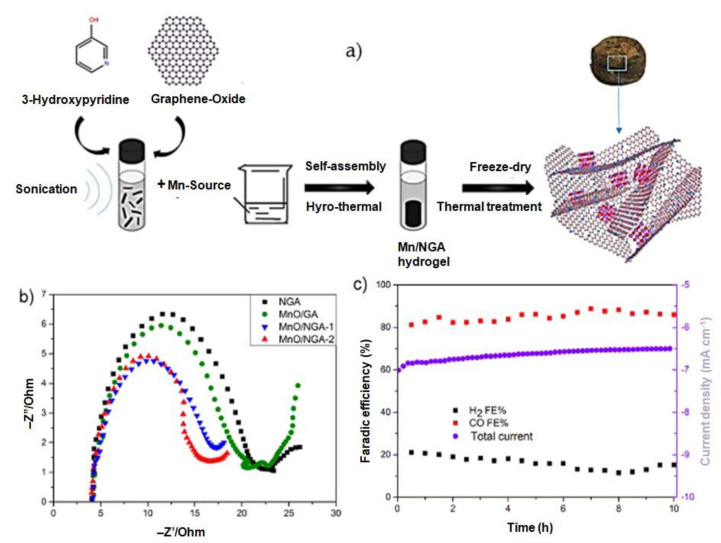
(**a**) Schematic representations of the fabrications of Mn-based heterogeneous catalysts, (**b**) Nyquist plots for NGA, MnO/GA, MnO/NGA-1, and MnO/NGA-2 catalysts, and (**c**) current-time plot and their Faradaic efficiencies for MnO/NGA-2. Reprinted with permission from [29]. The American Chemical Society Washington, DC, USA, 2020.

**Figure 5:**
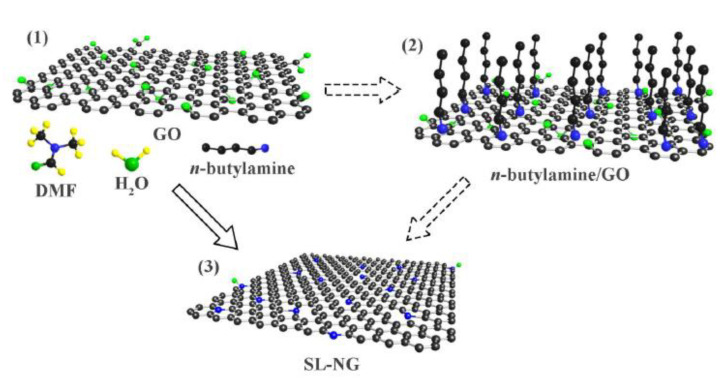
Schematic representation for the preparation of (1) single layer GO dispersion, (2) n-butylamine is adsorbed to GO layer; (3) SL-NG. Reprinted with permission from [30]. The American Chemical Society Washington, DC, USA, 2018.

Mengchu Wang [29] has successfully introduced a novel method for synthesizing a distinctive three-dimensional structure composed of nitrogen-doped graphene aerogel-supported MnO nanoparticles (MnO/NGA-1). This approach involves a series of steps, resulting in the formation of a unique hybrid material. This hybrid was created through a series of steps, including hydrothermal self-assembly, freeze-drying, and subsequent heat treatment techniques (Figure 4a). Figure 4b shows the Nyquist plots measured for four different systems, accompanied by the corresponding equivalent circuit of electrochemical impedance spectroscopy (EIS). Among these systems, the MnO/NGA-2 configuration displays the lowest charge transfer resistance, denoted by the radius of the semicircle (6.33 Ω). In comparison, the charge transfer resistance values for NGA, MnO/GA, and MnO/NGA-1 are 9.69 Ω, 8.32 Ω, and 6.64 Ω, respectively. The reduced charge transfer resistance observed in the MnO/NGA-2 electrode can be attributed to the incorporation of nitrogen into the graphene network. Figure 4c demonstrates that the MnO/NGA-2 system maintains a consistent current density of approximately 7 mA cm^−2^ throughout the duration of the test, with only minimal deviation after continuous testing for 10 h. To evaluate the Faradaic efficiency (FE), the concentration of gaseous products was periodically measured every 30 min. The FE of CO exhibits limited variability, remaining stable at around 86% throughout the 10 h electrolysis period. An in-depth examination was conducted on the nitrogen-doped graphene/graphdiyne heterostructure (TM-N@GRA/GDY) in order to analyze its potential as a single-atom catalyst for CO_2_ electroreduction applications, specifically focusing on the transition metal (TM) anchored on the structure. The computational findings indicate that Co-N@GRA/GDY demonstrates exceptional reactivity, displaying a remarkable ability to efficiently reduce CO_2_ to CH_4_. Notably, this catalyst exhibits an impressively low limiting potential of −0.567 V for the CO reduction process [46]. By utilizing a unique and straightforward one-pot synthesis approach, Huang et al. [30] have successfully produced a catalyst consisting of partially oxidized 5 nm cobalt nanoparticles dispersed on a single layer of nitrogen-doped graphene, denoted as PO-5 nm Co/SL-NG (Figure 5). This catalyst has proven to be highly effective for the electrocatalytic reduction of carbon dioxide to methanol under mild conditions in a 0.1 mol dm^−3^ aqueous NaHCO_3_ medium. Impressively, it achieves a maximum faradic efficiency of 71.4% for methanol at a low potential of −0.90 V vs. SCE. It also exhibits a robust electrocatalytic current density of 4 mA cm^−2^ and a high yield of 1.10 mmol dm^−3^ h^−1^, with an overpotential as low as 280 mV. Furthermore, at −1.0 V vs. SCE, a significant current density of 10 mA cm^−2^ can be attained, while maintaining a notable faradic efficiency of 23.2% for methanol. Noteworthy is the catalyst’s exceptional stability, as evidenced by its morphology, particle size, structure, and elemental composition remaining nearly unchanged even after 10 h of CO_2_ electroreduction. A highly efficient nanocomposite with excellent catalytic properties for the reduction of CO_2_ has been developed. This unique composite comprises Cu nanoparticles (NPs) and reduced graphene oxide (rGO) supported on a Cu substrate. Through extensive optimization, the compositions of Cu NPs and rGO, as well as the overall quantity, have been fine-tuned to achieve maximum performance. The optimized nanocomposite exhibits remarkable catalytic activity, demonstrating its ability to effectively convert CO_2_ into CO, HCOOH, and CH_4_. Furthermore, it showcases an impressive Faradaic efficiency (FE) of 76.6% when operated at −0.4 V (vs. RHE) in a NaHCO_3_ solution saturated with CO_2_. This Cu-rGO nanocomposite offers exceptional stability, further enhancing its potential for electrochemical CO_2_ reduction [47]. Graphene-based electrode materials offer numerous advantages, including high electrical conductivity, large surface area, mechanical strength, and chemical stability. However, they also come with disadvantages such as synthesis challenges, aggregation issues, and cost considerations. Researchers continue to work on addressing these challenges to unlock the full potential of graphene-based materials in various applications.

## 4. Metal-Oxide-Based Electrode Materials

Superior electrode architectures play a crucial role in enhancing the overall device performance of metal oxide nanostructures, which exhibit high specific capacity/capacitance, typically exceeding that of carbon/graphite-based materials by 2–3 times. Nevertheless, the current stability during cycling and rate performance of these nanostructures falls short of meeting practical application requirements. Hence, there is an urgent need to enhance their electrochemical properties by not only advancing the development of electrode materials but also focusing on the design aspect of superior electrode architectures [48,49]. Metal oxides play a significant role in CO_2_ reduction reactions due to their unique properties and catalytic capabilities. These oxides act as catalysts or catalyst supports in electrochemical systems, facilitating the conversion of CO_2_ into valuable products through reduction reactions. One key aspect of metal oxides is their ability to provide active sites for CO_2_ adsorption and subsequent reaction. The metal cations present in the oxide lattice can interact with CO_2_ molecules, promoting their activation and facilitating the reduction process. This interaction helps in overcoming the high energy barrier associated with CO_2_ reduction and enhances the efficiency of the reaction [50,51,52]. Cu_2_O-derived copper electrodes efficiently reduced carbon dioxide, yielding ethylene (C_2_H_4_) and ethanol as the primary C2 products. Boon Siang Yeo et al. [53]. reported optimized faradic efficiencies of 32.1% and 16.4% at −1.0 V vs. RHE for ethylene and ethanol, respectively. The catalysts responsible for this performance were CuO particles, around 500 nm in size, formed through the reduction of Cu_2_O during the initial phase of the CO_2_ reduction reaction. The addition of palladium(II)chloride to the electrolyte facilitated the production of C_2_H_6_ with a notable faradic efficiency of 30.1% at the mentioned potential. However, the formation of C_2_H_4_ was significantly suppressed, resulting in a faradic efficiency of only 3.4%. The surface of the BDD electrode was modified by applying iridium oxide, and its impact on CO_2_ electrochemical reduction was investigated. The modification resulted in a catalytic effect, leading to a reduction in the potential required for CO_2_ reduction.

**Figure 6:**
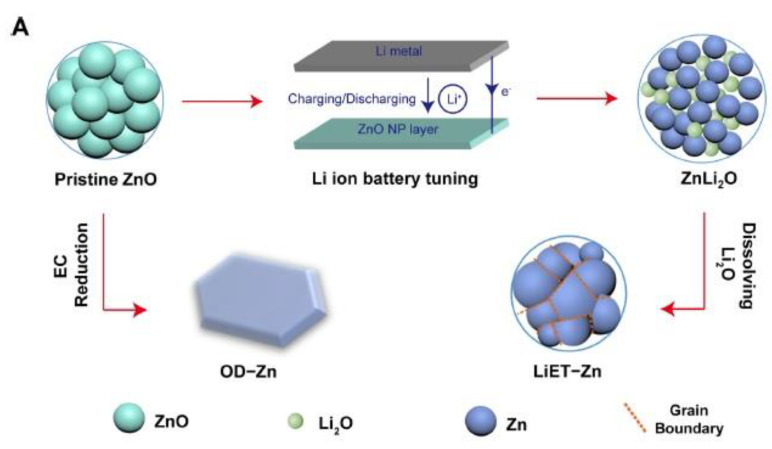
(**a**) Illustration depicting the structural transformation of ZnO through various reduction techniques and the voltage response during Li-assisted electrochemical tuning and (**b**) durability assessment of the electrocatalytic CO_2_ reduction reaction (CO_2_RR) utilizing LiET-Zn supported on a glassy carbon electrode in a long-term stability test in a CO_2_-saturated 0.1 M KHCO_3_ solution at –1.17 V vs. RHE with a loading of 0.2 mg cm^−2^ of LiET-Zn. Reprinted with permission from [56]. The American Chemical Society Washington, DC, USA, 2017.

**Figure 7:**
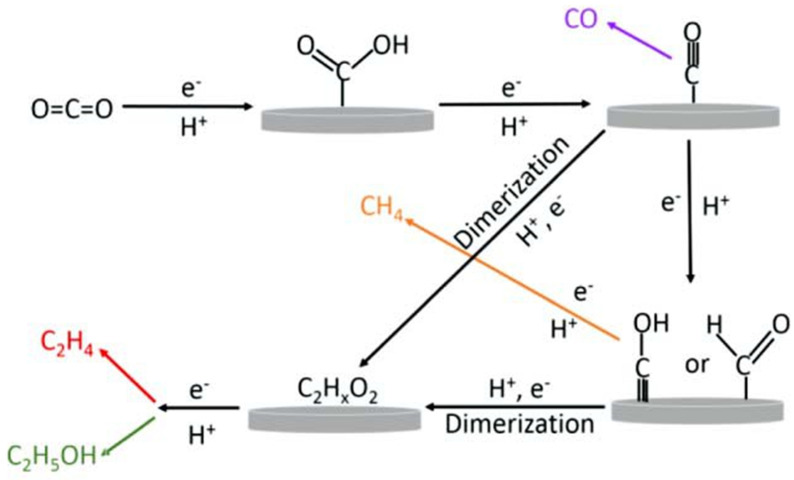
The proposed schematic representation of the electroreduction of CO_2_ to ethylene and ethanol on copper electrode. Reprinted with permission from [57]. The American Chemical Society Washington, DC, USA, 2015.

Additionally, when comparing the modified iridium oxide-coated BDD electrode to the unmodified BDD electrode at the same reduction potential, a higher efficiency in formate production was observed. These findings indicate that the modification enhances the electrocatalytic activity of the BDD electrode for CO_2_ electrochemical reduction [54]. Cu and Zn nanoparticles were prepared using the co-precipitation method and supported on N-doped graphene (CuZnx/NGN). Optimized ZnO loading was achieved to enhance electrochemical CO_2_ reduction (ECR) efficiency, resulting in the production of multi-carbon compounds. Notably, the CuZn20/NGN electrode demonstrated exceptional stability for at least 24 h under optimized conditions. This improvement is attributed to CO generation at the reduced ZnO nanoparticles, leading to increased *CO coverage on the reduced CuO. Consequently, the rate of C-C coupling is enhanced, facilitating the generation of multi-carbon compounds [55]. By utilizing the Li electrochemical tuning (LiET) method, we achieved a remarkable CO faradaic efficiency (FE) of up to 91.1% at an electrode potential of −1.17 V versus reversible hydrogen electrode (RHE), effectively suppressing H_2_ evolution. This performance stands out as one of the most exceptional results reported for CO_2_RR on zinc-based catalysts (Figure 6a). Additionally, we observed a gradual increase in overall current density from 26.5 to 30 mA cm^−2^ mg^−1^ during continuous 4 h electrolysis at −1.17 V versus RHE, accompanied by a sustained CO FE exceeding 80%. These outcomes highlight the excellent stability of the LiET-Zn catalysts, which, combined with their remarkable CO FE, position them as promising candidates for efficient CO_2_ reduction and renewable energy applications (Figure 6b) [56]. Boon Siang Yeo and co-authors [57] explored the electroreduction of CO_2_ using Cu_2_O films on electrodes at different potentials. By adjusting the thicknesses of the Cu_2_O overlayers, they were able to control the production of C_2_ products. The most efficient C-C bond formation occurred in films with thicknesses between 1.7 and 3.6 μm, resulting in the generation of ethylene and ethanol. The faradic efficiencies for ethylene and ethanol were in the range of 34–39% and 9–16%, respectively, while the formation of methane was greatly suppressed (faradic efficiency < 1%). This study achieved an unprecedentedly high ratio of C_2_H_4_ to CH_4_ products, approximately 100. A mechanistic model presented in Figure 7 illustrates the successive steps of the electrochemical reduction process, involving proton and electron transfer, formation of *COOH, hydrogenation to *CO, and subsequent reduction to ethylene or ethanol. Metal-oxide-based electrode materials offer advantages such as high specific capacity, chemical stability, and abundant resources. However, they also have limitations like limited electrical conductivity, volume expansion issues, and potential toxicity concerns. Researchers continue to explore strategies to improve the performance and address the disadvantages of metal-oxide-based electrode materials in various applications.

## 5. MOF-Based Electrode Materials

Over the past few decades, metal–organic frameworks (MOFs) have become increasingly prominent as a unique category of porous materials, showcasing exceptional surface and structural characteristics. This has propelled the utilization of MOFs in diverse applications such as drug delivery, gas separation and storage, catalysis, and sensors. Additionally, their impressive conductivity properties have positioned MOFs as highly efficient active substances in energy storage devices. Consequently, MOFs have generated significant attention in the fields of high-energy-density rechargeable batteries and supercapacitors [58,59,60]. Hyung Mo Jeong [61] proposes a technique to mitigate the impact of Fe impurities on Cu surfaces using nano-sized metal–organic frameworks (MOFs). By growing Zr-based MOFs (UiO-66) preferentially on Fe sites via a TPA-Fe coordination bond, the UiO-66@Cu film exhibits a significantly improved hydrocarbon Faradaic efficiency (FE) of 37.59% compared to 14.68% FE on a commercial Cu film. The method effectively suppresses the hydrogen evolution reaction (HER). XPS analysis shows selective binding of UiO-66 ligands to metallic Fe sites while leaving the metallic Cu unaffected, providing active Cu sites for CO_2_RR and enabling highly efficient hydrocarbon production. A zinc-based metal–organic framework (MOF) was synthesized using adipic acid via solvothermal and sonochemical methods. The research highlights the potential of MOFs in the circular economy, promoting energy recovery, waste reduction, and responsible resource consumption. These findings open avenues for cost-effective transitions to sustainable industries [62]. A novel framework, denoted as V12, was successfully constructed and characterized. The framework consists of {(MeNH)[Bi(L)].4DMF.2H_2_O}, where L represents 5,5′-(1,3,6,8-tetraoxo-1,3,6,8-tetrahydrobenzo[lmn][3,8]phenanthroline-2,7-diyl) dibenzene-1,3-dicarboxylic acid and DMF represents N,N-dimethylformamide. V12 exhibits large, one-dimensional channels with dimensions of approximately 1.5 × 0.7 nm and demonstrates excellent stability in common solvents. When V12 is modified on an electrode through electrodeposition, the resulting sample exhibits remarkable catalytic performance for CO transformation into formate. It achieves a Faraday efficiency of 93.2% and a current density of 11.78 mA cm at a potential of −0.9 V (vs. RHE) [63]. CuIn-MOF, a high-performance catalyst for electrochemical carbon dioxide reduction (eCO_2_R), demonstrates impressive Faradaic efficiency (FE) for CO and formate production. FE for CO reaches 78.6% at −0.86 V vs. RHE, while FE for formate is 48.4% at −1.16 V vs. RHE. These high FE values are maintained over a wide range of current densities (20.1–88.4 mA cm^−2^) and for a duration of 6 h, indicating excellent long-term stability. Anion-regulation engineering reveals the superiority of SO_4_^2−^ anion precursor for formic acid generation compared to NO^3−^. Additionally, Cu exerts a positive influence on eCO_2_R towards CO production when combined with SO_4_^2−^ anion precursor [64].

**Figure 8:**
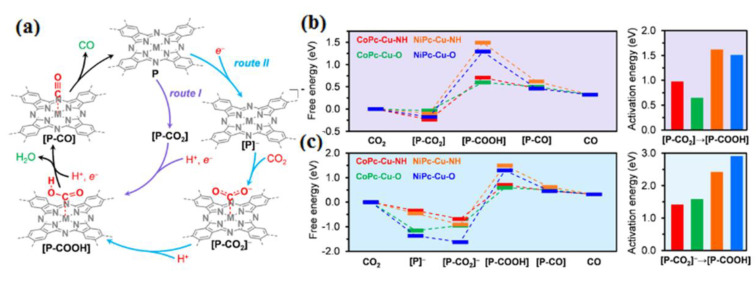
(**a**) Proposed catalytic mechanism for the electrochemical conversion of CO_2_ into CO using MP cactive sites within the framework of MPc-Cu-XH MOFs, with consideration of two distinct reaction pathways: Route I and Route II. Free energy profiles illustrating the electrochemical CO_2_-to-CO reduction catalyzed by CoPc-Cu-NH (in red), CoPc-Cu-O (in green), NiPc-Cu-NH (in orange), and NiPc-Cu-O (in blue) under standard conditions and an electrode potential of 0 V (relative to the standard hydrogen electrode) along the (**b**) Route I and (**c**) Route II reaction pathways. Reprinted with permission from [65]. The American Chemical Society Washington, DC, USA, 2020.

**Figure 9:**
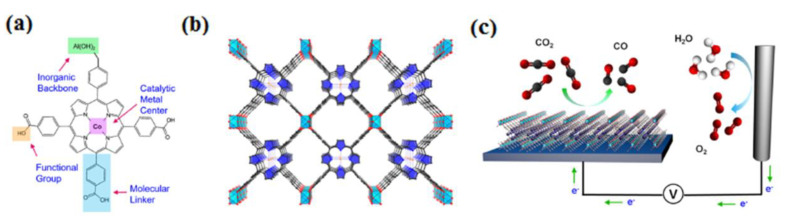
Our MOF catalyst enables precise control over metal centers, organic linkers, and functional groups at the molecular scale (**a**). The organic building blocks, represented by cobalt-metalated TCPP, are organized into a three-dimensional MOF structure, denoted as Al_2_(OH)_2_TCPP–Co, featuring adaptable inorganic building units (**b**). Within this arrangement, Co is depicted as orange spheres, O as red spheres, C as black spheres, N as blue spheres, Al as light-blue octahedra, and pyrrole rings as blue elements. Each carboxylate group from component A forms a bond with the aluminum inorganic framework. The MOF is integrated with a conductive substrate to create a functional electrochemical CO_2_ reduction system (**c**). Reprinted with permission from [66]. The American Chemical Society Washington, DC, USA, 2015.

Katherine A. Mirica conducted an experiment using 2D metal–organic frameworks (MOFs) made of metallophthalocyanine (MPc) ligands linked by copper (Cu) nodes. These MOFs had electrical conductivities ranging from 2.73 × 10^−3^ to 1.04 × 10^−1^ S cm^−1^ for the electrochemical reduction of carbon dioxide (CO_2_) to carbon monoxide (CO). The performance of these MOFs as catalysts depended on two crucial factors: the choice of metal (cobalt or nickel) within the MPc catalytic subunit and the type of heteroatomic cross-linkers (oxygen or nitrogen) between the subunits. The choice of metal in the MPc subunit played a dominant role in determining activity and selectivity, while the heteroatomic linkers further influenced the outcomes. The CoPc-Cu-O framework exhibited the highest selectivity for CO production (FECO = 85%) at a low overpotential of −0.63 V. When combined with carbon black at a 1:1 mass ratio, it also achieved high current densities of up to −17.3 mA cm^−2^. Comparative experiments with metal-free phthalocyanine MOF analogs supported the importance of the central metal within the phthalocyanine for catalytic activity, rather than the Cu nodes. Density-functional theory calculations showed that CoPc-based and O-linked MOFs had lower activation energies for carboxyl intermediate formation compared to NiPc-based and NH-linked analogs. These findings correlated with the higher activities and selectivity observed in the CoPc-based and O-linked MOFs. In this study, the authors highlighted the significance of metal choice within the MPc subunit and the type of heteroatomic linkers in determining the catalytic performance of conductive 2D MOFs for CO_2_ reduction. The CoPc-Cu-O MOF emerged as a promising catalyst, exhibiting high selectivity for CO production and substantial current densities at a low overpotential (Figure 8) [65]. The electrochemical reduction of aqueous CO_2_ was recently demonstrated to be applicable using MOF-integrated catalytic systems, showcasing their versatility as modular platforms. This study signifies the initial advancement in developing CO_2_ reduction electrocatalysts based on MOFs. The selection of the active site, inorganic backbone, thickness/loading, and subsequent integration onto a conductive support were all carefully chosen in this first-generation design. These systems offer modularity, presenting numerous prospects for enhancing performance and exploring novel directions in the field of electrocatalysis. The strategy employed for the electrochemical reduction of CO_2_ using MOFs involved the careful selection of MOFs containing catalytic linker components. These MOFs were then transformed into thin films that were applied to conductive substrates (refer to Figure 9). The first step in this process entailed assembling suitable catalytic linker units (depicted in Figure 9A) to form a porous thin film MOF. The subsequent stage involved the growth of this MOF on a conductive substrate, as illustrated in Figure 9C. Additional details and visual representations can be found in Figure 9B, showcasing the structure and characteristics of the porous thin film MOF [66]. Coatings derived from MOFs, when applied to copper surfaces, exhibit enhanced selectivity for the production of isopropanol compared to uncoated copper surfaces. Moreover, these electrodes coated with MOF-derived materials demonstrate the capability to generate higher current densities, thus presenting promising prospects for enhancing the efficiency of stacked cell electrolyzers [67].

MOFs offer advantages such as high surface area, tailorable properties, and versatility in various electrochemical applications. However, they also have limitations, including limited electrical conductivity, stability challenges, and potential toxicity concerns. Researchers continue to work on addressing these disadvantages and exploring the full potential of MOFs in electrode materials for energy storage and catalytic applications.

## 6. Metal-Based Electrocatalysts

**Figure 10:**
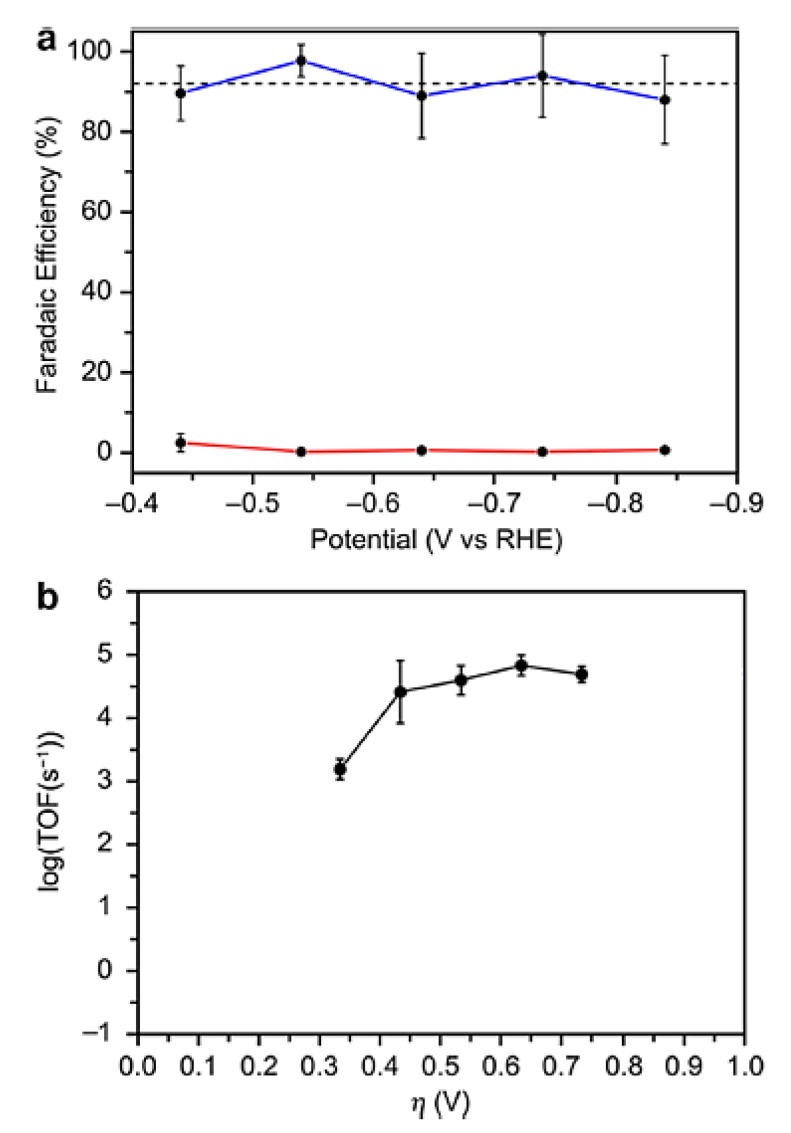
Performance of the Fe-tpyPY2Me electrocatalyst in the reduction of aqueous CO_2_. (**a**) Faradaic yields for CO production (in blue) and H_2_ generation (in red) under different applied potentials during a 60 min period (average values from three experiments). The mean FECO is highlighted with a dashed black line. (**b**) Tafel plot illustrating the catalytic activity of [Fe1]^2+^ derived from constant potential electrolysis (CPE) measurements in a 0.10 M NaHCO_3_ solution. Reprinted with permission from [76]. The American Chemical Society Washington, DC, USA, 2020.

With the changing global energy landscape, governments and the people of all countries are becoming more conscious of the importance of participating in the environmental governance system. The most critical of these is that the consumption of traditional fossil fuels has a vicious proportion with the glasshouse gas that the current environment has undertaken, inevitably causing an energy crisis, climate change, and a series of environmental questions [68]. People have begun to examine turning renewable electricity into chemical energy with storability, transportability, increased energy density, and enhanced safety in order to rationalize the application of power resources and address the current environmental issue. As an example, the commercial use of electrochemical water splitting technology to make hydrogen has effectively completed the process of converting renewable energy into a fuel production economy [69]. Gu’s group, for example, used a simple solid-state approach to create a “3D” electrocatalyst with first-rate activity, which enabled the efficient conversion of electrical energy to hydrogen energy in both acidic and alkaline electrolytes [70]. Sousa et al. [71] investigated the effect of cell design, process parameters, and GDE (gas diffusion electrode) composition on CO_2_ electrochemical reduction. The authors were able to produce one of the most stable cell performances documented, with an extraordinarily high FE (faradaic efficiency) of 85%, by adopting a flow-through-based Cu-GDE cell configuration. A one-step electrodeposition technique of Cu metal on a three-dimensional carbonaceous membrane produced four separate 4 cm -sized nanostructured Cu-based electrocatalysts. The Cu_2_O-Cu^0^ catalyst demonstrated the greatest electrocatalytic activity at a relatively low potential (0.4 V vs. RHE), with a consistent and low current density of 0.46 mA cm^−2^ and a productivity of 308 µmol g_cat−1_ h^−1^. Nielsen et al. used density functional theory to explore the aqueous-phase reduction of carbon dioxide by cobalt porphine, including the B3LYP, PBE, and BP86 techniques. For low-lying electronic states of cobalt porphines, which are probable intermediates in the reduction pathway, optimal structures and harmonic vibrational frequencies were determined. There were both planar and nonplanar cobalt porphine structures discovered; a nonplanar, ruffled structure was discovered for the ground state of [CoP]^+^, which has the lowest Co-N distance (1.947) of the porphine species studied [72]. Biological, heterogeneous, and molecular catalysts have all been investigated for CO_2_ reduction, with molecular systems in particular offering potential advantages such as their small size relative to enzymes and the ability to be tuned with atomic precision that heterogeneous materials do not [73,74,75]. Derrick et al. [76] described a molecular iron (II) system that embodies this design approach in a homogenous context by employing a redox non-innocent terpyridine-based pentapyridine ligand (tpyPY2Me). Because of the strong metal ligand exchange coupling between the Fe (II) center and ligand, [Fe(tpyPY2Me)]^2+^ exhibits redox behavior at potentials 640 mV higher than the isostructural [Zn(tpyPY2Me)]^2+^ analog, which contains the redox-inactive Zn (II) ion. The variable potential CPE tests with direct product detection reveal that [Fe1]^2+^ functions at fast rates in both organic (TOF > 100,000 s^−1^) and aqueous electrolytes (TOF > 50,000 s^−1^) while retaining strong long-term stability and recyclability (Figure 10).

Qiuet al. [77] showed that incorporating Cu-MOF into a Cu-nanoparticle-based GDE (gas diffusion electrode) may significantly improve the selectivity of CH_4_ for the electrochemical reduction of CO_2_, in addition to a higher positive onset potential and outstanding durability. The synthesized Cu-MOF showed significantly improved CO_2_ capture performance, allowing it to continuously provide CO_2_ for subsequent ERC reactions and favor the creation of ERC (electrochemical reduction of carbon dioxide) products. CO_2_ reduction on nanostructured WO_3_ electrodes in both dry and wet ACN solutions was studied using electrochemical and spectroelectrochemical measurements. CO_2_ reduction begins at −0.16 V versus NHE, indicating that WO_3_ can operate as an electro catalyst for CO_2_ reduction. However, current densities at potentials significantly negative to the onset potential are small, owing to the mechanism involving WO_4_^2−^ unit dissolution/deposition [78]. By comparing the results obtained with flat Au and nanostructured Au electrodes conducted in two different electrolytes, KHCO_3_ and NaHCO_3_, Kim et al. demonstrated how the electrode catalyst surface structure and the electrolyte composition influence the efficiency and stability of the CO_2_ reduction reaction. The study results showed that CO_2_ reduction to CO production efficiencies were improved in the KHCO_3_ electrolyte with both flat and nanostructured Au catalysts, with the flat Au surfaces showing signals of greater improvement in the first period. Because of their low hydration capabilities, bigger cations have a higher propensity for adsorption on the Au surface, resulting in increased CO FE in the KHCO_3_ electrolyte [79]. The boron-doped diamond (BDD) electrodes produced a large amount of formic acid and a tiny amount of carbon monoxide. When reducing CO_2_ with BDD electrodes, switchable product selectivity was accomplished [80]. According to Ramli et al. [81], carbon as a support electrode may improve mass transfer, conductivity, active surface area, and even metal stability decorated on the surface of the carbon-based electrode. The self-templated hollow Cu/CeO_2_ nanotubes exhibit a high faradaic efficiency (FE) of 78.3% for the electrochemical reduction of CO_2_ into ethylene (C_2_H_4_) in flow cell at a low applied potential of −0.7 V vs. RHE. The remarkable reduction efficiency of Cu/CeO_2_ nanotubes catalyst can be due to synergistic effects caused by the creation of an inseparable interface structure between Cu and CeO_2_, which improves the efficient adsorption of intermediates [82]. Metal-based electrocatalysts offer advantages like high catalytic activity, versatility, and durability, making them essential components in various electrochemical processes. However, they also have limitations, including cost (for precious metals), susceptibility to poisoning, and potential environmental concerns. Researchers continue to explore new catalyst materials and design strategies to overcome these limitations and improve the efficiency and sustainability of electrochemical processes.

## 7. Transition-Metal-Based Electrocatalyst

**Figure 11:**
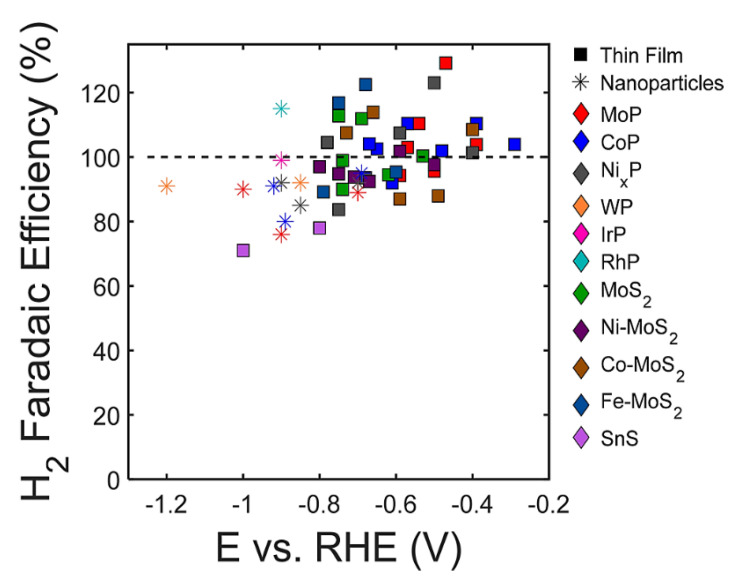
Faradaic efficiency for hydrogen (H_2_) generation in the context of CO_2_ reduction (CO_2_R) conditions for select transition metal phosphide and sulfide materials. Reprinted with permission from [87]. The American Chemical Society Washington, DC, USA, 2018.

Numerous carbon capture and storage (CCS) and carbon capture and utilization (CCU) methods have been developed to minimize carbon dioxide (CO_2_) emissions from hazardous gases generated in power generating and other industrial production operations. Electrochemical CO_2_ reduction is regarded as one of the most promising CCU techniques since it operates in moderate conditions, requires extremely basic and modest plant footprints, and can be created using modular and scalable equipment. Catalysts play crucial roles at both the cathode and anode in order to produce a suitable rate of CO_2_ reduction at viable overpotentials by optimizing the reaction pathways’ free energy environment. A high overall reaction rate and good selectivity of the CO_2_R product necessitate the use of an efficient and effective cathode catalyst to reduce CO_2_ [83]. Kang et al. [84] use first-principles calculations to study the CO_2_R activities of anion vacancies in two-dimensional (2D) transition-metal dichalcogenides (TMDs) and identify ReS_2_ and ReSe_2_ as interesting candidates with a low onset potential for CO_2_R and high selectivity versus the HR. When the Co_3_O_4_/CuO mixed-oxide nanosheets were used for methanol oxidation and carbon dioxide (CO_2_) conversion, it was found that its activity was superior to that of the respective Co_3_O_4_ and CuO metal oxide NSs in both methanol oxidation and CO_2_ conversion. The combined Co_3_O_4_/CuO NS mass activity was 12 mA g^−1^, which is 2.4 times greater than that of the Co_3_O_4_ which has a mass activity of 5 mA g^−1^ and four times greater than that of the CuO NS, which has a mass activity of 3 mA g^−1^. This mass activity was created at 0.627 V versus Ag/AgCl during methanol oxidation (0.5 M) [85]. For the first time, Zu et al. colleagues used theoretical and electrochemical experimental research to provide inferential insight into the detection and quantification of CO_2_ in deep eutectic fluids. The researchers demonstrated electrochemical assessment of CO_2_ solubilities in newly produced transition-metal-based deep eutectic solvents (TDESs) for better CO_2_ sorption. The employment of a transition metal precursor as a hydrogen bond acceptor in the preparation of TDESs can result in additional CO_2_ sorption sites [86]. The link between the hydrogen evolution process (HER) and CO_2_ reduction (CO_2_R) on transition metal phosphide and transition metal sulfide catalysts has been studied using a combination of experiment and theory. In comparison to CO_2_R, each of the investigated catalysts has shown a high faradic efficiency for H_2_ evolution (Figure 11). Although the presence of multifunctional active sites in these materials may boost their CO_2_R activity compared to pure transition metal electrocatalysts, under aqueous testing circumstances, these materials demonstrated a high selectivity for the HER over CO_2_R [87].

By using the density-functional method, a number of transition-metal (M=Mn-Cu, Ru-Ag)-doped C_3_N monolayers (M-C_3_N) as new CO_2_ER catalysts have been studied. Owing to its strong catalytic activity and good selectivity, Mn-C_3_N has proven to be the optimum catalyst for CO_2_ER via accurate computational screening. The end-product, HCOOH, has a kinetic energy barrier of 0.75 eV and a low overpotential of 0.04 V. On the surface of Mn-C_3_N, the development of hydrogen evolution is likewise prevented. Therefore, the metal atom in the C_3_N monolayer may be changed to tune the CO_2_ERactivity, which may provide novel insight into the development of innovative C_3_N-based CO_2_ER catalyst [88]. Early transition metal complexes have been used to observe stoichiometric and catalytic reductions of CO_2_ to methanol and related products, and highly active catalysts based on these complexes may be created to reduce CO_2_ to liquid fuels like methanol or to hydrocarbons like methane [89]. The ionic liquid [Emim][TFA], with 33% water as a cosolvent, has been shown to be an effective medium for the conversion of carbon dioxide to formate. The formate concentration in the electrolysis mixture was directly detected using deuterium labelling and proven to be derived from carbon dioxide [90]. Copper (Cu)-based catalysts have been widely used in the electrochemical reduction of carbon dioxide (ERCD) due to their distinctive activity in the synthesis of alcohols and hydrocarbons. Cu foam has a three-dimensional (3D) porous structure and catalytically active Cu elements, making it an excellent catalytic material for ERCD. In situ fabrication of nanostructured self-supporting Cu electrodes with Cu foam as the substrate and successive morphologies of nanowires (CuNW), nanosheets (CuNS), and nanoflowers (CuNF) is accomplished by simply altering the reaction time in a strongly alkaline oxidizing solution. The morphology of the as-manufactured, nanostructured, self-supporting Cu electrodes and the electrolyte species affects the performance and product distribution of ERCD. Because of its nanosheet morphology, which can better stabilize the intermediate state products, the CuNS electrode has the highest Faradaic efficiency (FE) of 86.9% at −0.4 V (vs. RHE) and improved performance [91]. Finding an earth-abundant, highly active catalyst that selectively yields hydrocarbons at very low over potentials is a major issue for the electrochemical carbon dioxide reduction reaction (eCO_2_RR). The authors discuss the two-dimensional transition metal carbide class of materials’ eCO_2_RR performance. The research findings show that di-tungsten carbide (W_2_C) nanoflakes operate with a maximum methane (CH_4_) current density of about −421.63 mA/cm^2^ and a CH_4_ faradic efficiency of 82.7% ± 2% in a hybrid electrolyte of 3M potassium hydroxide and 2M choline-chloride [92]. Transition-metal-based electrode materials offer advantages like high catalytic activity, versatility, and durability, making them essential in various electrochemical applications. However, they also have limitations, including high cost, susceptibility to poisoning, and potential environmental concerns. Most of the researchers continue to explore new catalyst materials and design strategies to address these limitations and enhance the efficiency and sustainability of electrochemical processes.

## 8. Conducting Polymers

Conducting polymers, also known as intrinsically conducting polymers, are a class of polymer with superior electrical and optical properties that finds many applications in energy storage and conversion systems. CPs with appropriate electroactive characteristics have been used as effective catalysts for energy and associated environmental applications. Both electrochemical- and photoelectrochemical-based systems have been studied by a number of researchers for carbon dioxide reduction reactions (CRR). Recently, poly aniline–metal complexes for electrocatalytic CRR have been reported by Sassone et al. [93]. They have reported that the proposed catalyst is a binder-free superior elecrocatalyst, and in particular, Sn-Poly(aniline) exhibits excellent activity of 40% faradic efficiency compared to other metal polymer complexes. Diao et al. [94] showed a photocatalyst CO_2_ conversion of 3000 μmol g/h in less than 10 h using nanofibrillar poly(3,4-ethylenedioxythiophene) doped with hydrazine. They proposed that the band gap could be optimized via chemical doping of mineral acids (e.g., HCl), ammonium hydroxide, and hydrazine. Ponnurangam et al. [95] demonstrated that nitrogen-containing polymers with metals such as poly(aniline) and poly(pyrrole) exhibit excellent CRR. These polymers serve as direct catalysts as well as promoters and stabilizing agents. Recently, Hui and Luna summarized in detail a means improve the electrocatalytic performance of electroactive materials for CRR. They found that the proton and electron conducting electrodes and their materials are important to enhance the CRR. The conducting polymers, especially poly (aniline), poly(pyrrole), poly(3,4-ethylenedioxythiophene),have excellent catalytic conversion due to the presence of nitrogen-conducting polymers [96]. Composite electrodes made up of CuFe_2_O_4_@PANI exhibit better photo electrocatalytic responses towards CRR. They found that the polymeric and metal catalysts help to improve the physisorption of CO_2_ [97]. Alkaline (KOH)-treated PANI nanofibers were prepared via a one-step oxidative template assembly method to construct the N-doped hierarchical electrode. These electrodes showed a very good absorption of more CO_2_ capacity of 4.01 mmol/g at 25 °C under 1 bar. Additionally, these electrodes showed excellent selectivity of CO_2_ gas from the mixture of CO_2_/N_2_ [98]. The electrochemical simultaneous waste water treatment approach of electroreduction of CO_2_ at PR/PANI@ZnO paired with wastewater electro-oxidation over lead oxide electrode was demonstrated by Khalili et al. [99]. PANI with metals such as Pd, C, ZnO has received much attention due to their enhanced catalytic properties. The authors proposed that the use of ZnO would help to improve the amphoteric oxidation of CO_2_/CO with more electron transfer ability. This composite electrode showed an excellent catalytic response towards CRR. PANI date-seed-derived activated carbon PANI-DSAC has been developed and used to recover Fe^2+^ and Cu^2+^ from mining waste water through a capacitive deionization process. Furthermore, CuFe_2_O_4_/carbon fiber DSAC was fabricated via thermochemical conversion, and the resultant catalyst showed excellent photocatalytic reduction of CO_2_ to formic acid. The authors reported that the PANI-DSAC showed a bi-function activity and will be utilized for the waste treatment and waste utilization process [100].

**Figure 12:**
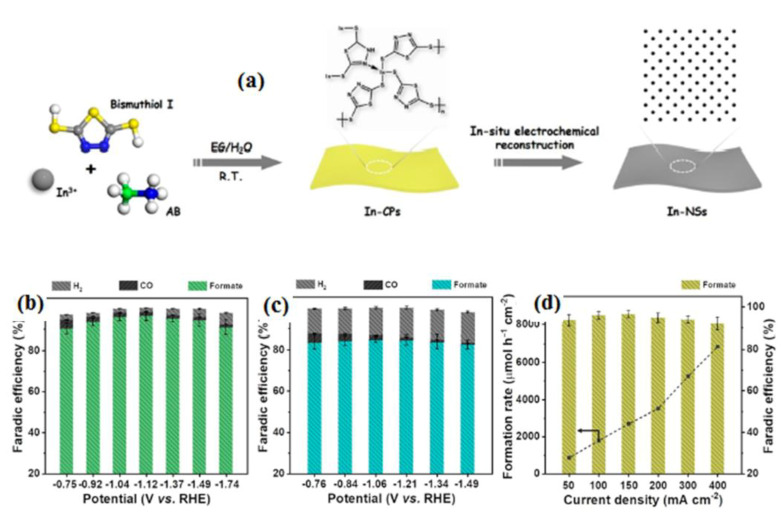
(**a**) Illustrative depiction outlining the process of synthesizing In-NSs from polymers and their subsequent CO_2_ reduction performance. Voltage-dependent Faradaic efficiencies (FEs) for formate and gaseous products for (**b**) In-NSs and (**c**) In-NPs. (**d**) The rate of formate product formation and FEs over In-NSs at different current densities. Reprinted with permission from [102]. The American Chemical Society Washington, DC, USA, 2023.

In another process, Yu’s group [101] developed an H_2_SO_4_ and ammonium-lauryl-sulfate-doped poly-aniline-based gas diffusion electrode for CRR. They indicated that the prepared electrode has increased biocompatibility, which resulted in faster start-up and higher bio-production of volatile fatty acts. The PANI-based electrode provides excellent absorptivity of CO_2_, and these bi-dopants provide superior ionic promoting ability for the absorption and conversion of value-added materials. Shao-HaiLi et al. [102] successfully overcame the challenge of synthesizing polymeric-derived 2D monometallic nonlayered indium through a simple electrochemical reduction approach (Figure 12a). In both In-NSs and In-NPs, the current density was observed to be higher in the presence of a CO_2_ atmosphere compared to the N_2_ atmosphere. This observation suggests that the CO_2_RR (carbon dioxide reduction reaction) is more favorable than the hydrogen evolution reaction process for these materials. The polymeric-derived In-NSs shows better formate formation selectivity and higher formate current density compared to sphere-like In-NPs (indium nanoparticles). The nanosheets have a greater tendency to produce formate and do so at a higher rate compared to the spherical nanoparticles. The unique structure and surface properties of In-NSs likely contribute to their enhanced performance in this specific reaction (Figure 12b,c). By performing calculations, the partial current density for formate at various potentials is determined. The most notable result is achieved at an applied potential of −1.74 V, where the highest formate current density of 360 mA cm^−2^ is obtained. Additionally, at this potential, the formate formation rate reaches an impressive value of over 6800 μmol h^−1^ cm^−2^. Moreover, the formate faradaic efficiency (FE) remains consistently high, exceeding 90% throughout these experiments (Figure 12d). Conducting polymer-based electrode materials offer advantages like tunable properties, flexibility, and low cost. However, they also have certain limitations, including limited electrical conductivity, stability issues, and electrode potential environmental concerns. Recently, many researchers have continued to explore new conducting polymers and improve their properties to expand their applications and address these limitations.

## 9. MXenen-Based Electrodes

Mxenes, a new class of two-dimensional transition metal carbides, nitrides, and their associated nanostructures, have been extensively studied as electrode materials for CRR. The general formula of Mn + 1XnTn (n = 1–4; M = transition metal; X = carbon and/or nitrogen; Tx = surface terminal group). Due to their unique electronic, mechanical, chemical, and physical properties with controlled surfaces with large surface areas, these classes of materials are promising high-performance electrocatalysts and utilized for energy storage and conversion applications [103]. Seh’s group have developed 2Dtitaniumand molybdenum carbide Mxenes and their associated materials are treated with HF and KF-HCl to enhance the hydrogen adsorption and surface etching process. Both theoretical and experimental studies have shown that the hydrogen evolution reaction is major barrier and necessitates a non-aqueous medium [104]. Baskaran and Jung investigated both theoretical and experimental studies on various transition-metal-anchored Mo_2_CS_2_, a single-atom-based heterogeneous catalyst, towards the electrochemical reduction of CO_2_. They found that the Fe, Co, Ni are effective towards C1 products and Fe; Co and Ru-supported catalysts selectively produce CH_4_. They reported that Ru@ Mo_2_CS_2_ has the lowest over potential of 0.24 V [105]. Again, Seh’s group has discussed the possibility of using Mxenes for the electrochemical reduction of CRR. A number of theoretical studies have been published on this topic and still, optimistic prospects for Mxenes as CRR remain unrealized. The linear scaling relationship of catalyst development and how the defect in Mxenes will help improve the catalytic response has been discussed in detail. The various bi-metallic and heterostructures have been discussed in a detailed manner [24,106].

**Figure 13:**
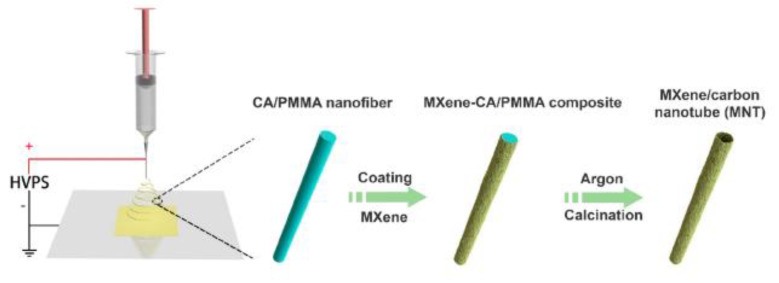
Schematic representation of the synthetic routes for MNT. Reprinted with permission from [108]. The American Chemical Society Washington, DC, USA, 2021.

Attanayake et al. [107] have developed 2D Mo_2_C and Ti_3_C_2_ Mxene-based electrocatalysts for CRR. The catalysts exhibit a Faradic efficiency for CO_2_ to CO of about 90% (Mo_2_C) and 65% (Ti_3_C_2_ Mxene) in acetonitrile using an ionic liquid—1-ethyl-2-methylimidazolium tetrafluoroborate electrolyte. Theoretical studies have revealed that the oxygen vacancies on both MXenes’ surfaces provide an activesites for the catalysis. A novel, two-dimensional Ti_3_C_2_Tx MXene/carbon heterostructure was successfully synthesized using a self-sacrificial templating method (Figure 13). This heterostructure demonstrated excellent electrochemical performance with an ultralow overpotential of 1.38 V at 0.2 A·g-1 in Li-CO_2_ batteries. The design of the parallel-aligned tubular architecture contributed to a high surface area, ensuring efficient CO_2_ adsorption and lithium carbonate decomposition. In situ and ex situ characterizations, along with density functional theory calculations, supported the enhanced catalytic performance and stability of the heterostructure. Moreover, the tubular architecture’s durability allowed for a long cycle life and facilitated proper interactions among gas, electrolyte, and electrode [108]. MXene-based electrodes offer advantages like high electrical conductivity, tunability, and chemical stability, making them promising materials for various applications. However, they also have limitations, including moisture sensitivity, synthesis complexity, and limited scalability. Ongoing research aims to address these challenges and unlock the full potential of MXenes in a wide range of technologies.

## 10. Free-Standing Electrode Materials

**Figure 14:**
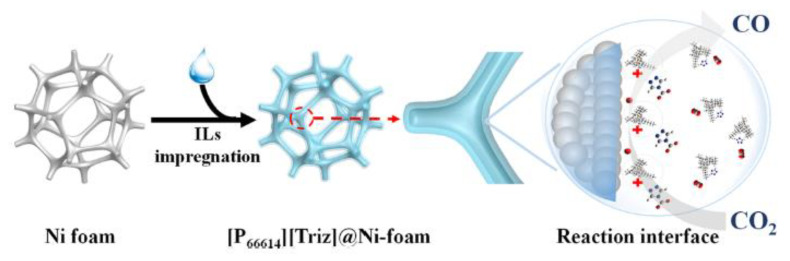
Diagram depicting the electrochemical reduction of CO_2_ on the [P66614][Triz]@Ni foam electrode. Reprinted with permission from [109]. The American Chemical Society Washington, DC, USA, 2022.

**Figure 15:**
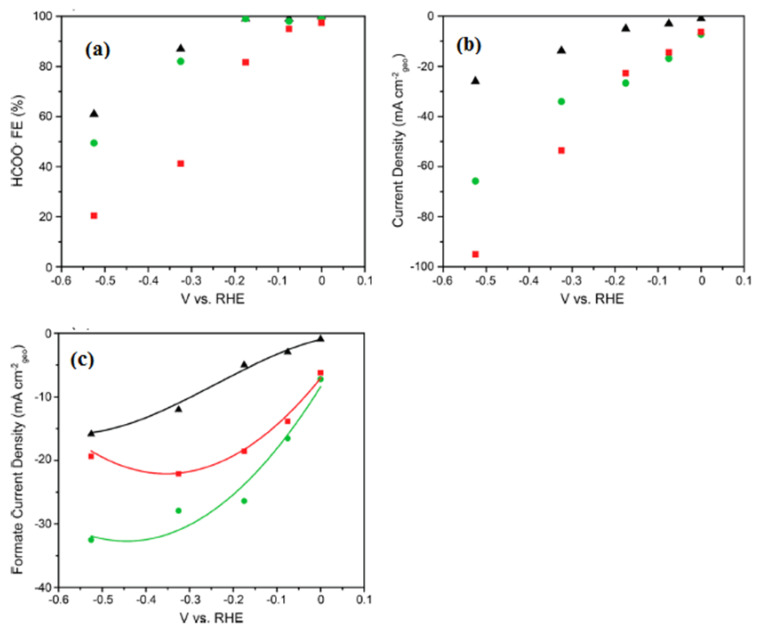
(**a**) HCOO^−^ Faradaic yield in relation to the electrolysis potential for CO_2_ reduction reactions (CO_2_RR) with Pd/C (depicted as black triangles), np-PdNi (shown as green circles), and np-PdCo (represented as red squares). (**b**) The steady-state current for CO_2_RR, normalized by the electrode’s geometric area, for Pd/C (black triangles), np-PdNi (green circles), and np-PdCo (red squares). (**c**) Partial current density for formate (HCOO-) production with Pd/C (black triangles), np-PdNi (green circles), and np-PdCo (red squares). All experimental data were collected in a CO_2_-saturated 1 M KHCO_3_ solution. Reprinted with permission from [110]. The American Chemical Society Washington, DC, USA, 2019.

Free-standing electrode materials for carbon dioxide (CO_2_) reduction have emerged as a promising avenue to tackle the global challenge of climate change and to create sustainable energy solutions. These materials are designed to efficiently catalyze the conversion of CO_2_ into valuable chemicals and fuels, thus mitigating the impact of greenhouse gas emissions on the environment. Here, we will delve deeper into some of the key aspects of these materials: Free-standing electrodes offer several advantages in CO_2_ reduction processes. Their independent, self-supporting nature ensures flexibility and ease of integration into various electrochemical setups, which can be particularly useful for large-scale applications. This electrode materials for CO_2_ reduction are an exciting area of research and innovation, offering a pathway to transform harmful greenhouse gases into valuable resources while contributing to a more sustainable and eco-friendly future [25]. In the realm of free-standing electrode materials for carbon dioxide reduction, there exists a scarcity of extensive literature on this specific topic. The available research and studies in this area are limited, posing challenges for comprehensive understanding and advancement in the field. Chuanxin He and colleagues [109] have introduced a simple and efficient approach to fabricate a free-standing electrocatalyst called [P66614][triz]@Ni foam. In this method, they confined a superbase and a hydrophobic ionic liquid (IL) called [P66614][triz] into the pores of Ni foam. This innovative technique allowed the ILs to modify the surface properties of the Ni foam, creating a microenvironment with a high concentration of CO_2_ near the electrode/electrolyte interface. The elucidated electrochemical carbon dioxide reduction (ECR) mechanism on an IL-modified Ni foam is depicted in Figure 14. Within IL mixed nonaqueous electrolytes, the initial step involves the diffusion of CO_2_ molecules from the bulk solution to the electrode/electrolyte interface. Subsequently, CO_2_ undergoes reduction, acquiring a single electron and transforming into the CO_2_^•−^ radical anion. The CO_2_^•−^ species then undergoes dimerization, resulting in the formation of oxalate as the product. During the reaction, CO_2_ can take two primary pathways: one involves the protonation of CO_2_^•−^ with water or another protic source, while the other involves the reaction of CO_2_^•−^ with another CO_2_ molecule, yielding carbonate and CO as products. Novel catalytic electrodes, namely np-PdX where X represents Ag, Cu, Ni, or Co, have undergone significant development. Among these, np-PdCo and np-PdNi have demonstrated remarkable attributes, exhibiting stable and selective electrolytic evolution of HCOO- from CO_2_ with impressive areal HCOO^−^ current densities surpassing 20 mA cm^−2^. In stark contrast, conventional Pd/C electrocatalysts, while proficient in generating HCOO- at low overpotential with high Faradaic efficiency (FE), suffer from rapid deactivation. This deactivation occurs despite efforts to maintain a low COOH_ad_/HCOO_ad_ intermediate favorability ratio, which is attributed to the substantial negative adsorption free energy of CO on Pd. In Figure 15a, we observe the potential-dependent Faradaic efficiency (FE) for HCOO- formation in a 1M KHCO_3_ solution, comparing Pd/C, np-PdNi, and np-PdCo. Pd-based materials exhibit nearly 100% FE for formate generation when operated at overpotentials below 200 mV, specifically in the potential range between 0 and −0.2 V vs. RHE. Interestingly, the FE trend for np-PdX closely resembles that of Pd/C within this specific potential range. Parts b and c of Figure 15 represent the total CO_2_RR constant potential electrolysis current density and formate partial current density, respectively [110]. Free-standing electrode materials offer advantages like high specific capacity, improved energy density, and enhanced mass transport. However, they also have limitations, including mechanical fragility, complex fabrication processes, and potential cost and environmental concerns. Researchers are actively working on addressing these challenges to unlock the full potential of free-standing electrodes in various electrochemical and energy storage applications.

## 11. Summary and Outlook

In the future, addressing environmental concerns and creating high-value fuels and chemicals will be crucial, and one way to achieve this is by integrating carbon dioxide molecules with renewable power systems. This approach also represents a strategic path towards attaining a sustainable and low-carbon economy. Emerging as a non-precious metal material, metallic Cu and its derivatives exhibit exceptional electrochemical stability and distinctive selectivity in producing high-value multi-carbon products. The mechanistic behavior of CO_2_ electrochemical reductions on various catalytic materials has been emphasized in our research. However, there is a need for further investigations that concentrate on the application of porous materials and delve into the CO_2_ adsorption and activation on the surfaces of heterogeneous catalysts. These aspects play a pivotal role in facilitating the subsequent reduction steps and should be the focus of future research endeavors.

Throughout this discussion, we have highlighted several cost and time-effective routes that possess environmentally friendly and energetically convenient characteristics. These advantageous features make them suitable for mass-scale material production. Consequently, these routes hold great promise for integration into large-scale CO_2_ electrolysis technologies, which can significantly enhance the widespread production of C_1_ chemicals. Copper catalysts show great potential in electrochemical CO_2_ reduction; however, achieving low-cost and scalable deployment of CO_2_ reduction technologies requires significant further research. To make progress in this direction, it is essential to enhance both our fundamental understanding of CO_2_ reduction mechanisms and practical performance metrics. Gold- and silver-based materials are the most active and selective catalysts for CO production. Recently, it has been demonstrated that single-atom catalysts possess high catalytic activity, selectivity, and stability for the electrocatalytic reduction of CO_2_ to CO. The development of catalyst design strategies has included reducing the size to the nanoscale level, exposing specific facets, quantum confinement, doping, alloying, and defect engineering to enhance the performance of the catalysts in CO_2_ reduction by modifying their electronic and geometric structures. We will now briefly outline our perspective on crucial areas for future investigations in these domains. In conclusion, there are still numerous challenges and opportunities to address in the development of catalysts and reactor systems with excellent activity, selectivity, stability, and scalability. Nevertheless, significant strides have been made in recent years, positioning this technology for potential commercial implementation.

## Figures and Tables

**Table 1:** Shows the different kinds of electrocatalyst for CO_2_RR products with their corresponding current density and Faradaic efficiency.

S.No	Electrode	Product	Electrolyte (M)	Current Density (mA cm^−2^)	Faradaic Efficiency (%)	Ref.
1	Cu/Cu_2_O	Ethylene	1 KCl	200	84.5	[11]
2	Sb-SnS_2_nanosheets	Formate	0.1 KHCO_3_	17.17	90.86	[15]
3	Ni-rich (Pd_20_-Ni_80_/ZC)	Carbon monoxide	1 KOH	200	95.3	[20]
4	Cu NPs	Ethylene	1 KOH	300	35.0	[22]
5	3D net-like CoSA/HCNFs nanofibers	Carbon monoxide	0.1 KHCO_3_	67	91.0	[25]
6	N-Doped Nanoporous Carbon	Carbon monoxide	0.5 NaHCO_3_	−0.40	90.0	[26]
7	Ni_3_N/MCNT	Carbon monoxide	0.5 NaHCO_3_	6.5	89.0	[27]
8	N-C-CoPc NR	Carbon monoxide	0.1 KHCO_3_	30	85.3	[28]
9	MnO/NGA-1	Carbon monoxide	0.5 KHCO_3_	-	86.0	[29]
10	PO-5 nm Co/SL-NG	Methanol	0.1 NaHCO_3_	10	71.4	[30]

1 Copper oxide nanoplate; 2 antimony-doped tin disulphide; 3 mixed-metal-oxide-based zeolite composite; 4 copper nanoparticles; 5 single-atom-cobalt-site-based high-yield carbon nanofiber; 6 nitrogen-doped nanoporous carbon; 7 nickel-nitride-based multi-walled carbon nanotubes; 8 cobalt phthalocyanine anchored by an N-doped porous carbon nanorod (N-C-CoPc NR); 9 MnO nanoparticles on three-dimensional N-doped graphene aerogels (NGAs); 10 partially oxidized 5 nm cobalt nanoparticles dispersed on single-layer nitrogen-doped graphene.

## Data Availability

Not applicable.

## Abbreviations

CRR	Carbon dioxide reduction reaction
eCO2R	Electrochemical carbon dioxide reduction
CNT	Carbon nanotube
MWCNT	Multi-walled carbon nanotube
MOFs	Metal-organic frameworks
CCS	Carbon capture and storage
CCU	Carbon capture and utilization
TMD	Transition metal dichalcogenides
PANI	Polyaniline
